# A Bibliometric Analysis of Pyroptosis From 2001 to 2021

**DOI:** 10.3389/fimmu.2021.731933

**Published:** 2021-08-18

**Authors:** Dan Ma, Bin Yang, Baoyi Guan, Luxia Song, Qiyu Liu, Yixuan Fan, Lin Zhao, Tongxin Wang, Zihao Zhang, Zhuye Gao, Siming Li, Hao Xu

**Affiliations:** ^1^Xiyuan Hospital, China Academy of Chinese Medical Sciences, Beijing, China; ^2^Graduate School, Beijing University of Chinese Medicine, Beijing, China; ^3^National Clinical Research Center for Chinese Medicine Cardiology, Xiyuan Hospital, China Academy of Chinese Medical Sciences, Beijing, China

**Keywords:** pyroptosis, CiteSpace, VOSviewer, programmed cell death, NLRP3, GSDMD, caspase

## Abstract

**Background:**

Pyroptosis is a new programmed cell death discovered in recent years. Pyroptosis plays an important role in various diseases. Nevertheless, there are few bibliometric analysis systematically studies this field. We aimed to visualize the research hotspots and trends of pyroptosis using a bibliometric analysis to help understand the future development of basic and clinical research.

**Methods:**

The articles and reviews regarding pyroptosis were culled from Web of Science Core Collection. Countries, institutions, authors, references and keywords in this field were visually analyzed by using CtieSpace and VOSviewer software.

**Results:**

A total of 2845 articles and reviews were included. The number of articles regarding pyroptosis significantly increased yearly. These publications mainly come from 70 countries led by China and the USA and 418 institutions. We identified 605 authors, among which Thirumaladevi Kanneganti had the most significant number of articles, and Shi JJ was co-cited most often. *Frontiers in immunology* was the journal with the most studies, and *Nature* was the most commonly cited journal. After analysis, the most common keywords are nod like receptor family pyrin domain containing 3 inflammasome, apoptosis, cell death, gasdermin D, mechanism, caspase-1, and others are current and developing areas of study.

**Conclusion:**

Research on the pyroptosis is flourishing. Cooperation and exchanges between countries and institutions must be strengthened in the future. The related pathway mechanism of pyroptosis, the relationship between pyroptosis and other types of programmed cell deaths as well as the role of pyroptosis in various diseases have been the focus of current research and developmental trends in the future research.

## Introduction

The term of “pyroptosis” was first proposed in 2001 by American scholars Cookson and Brennan (1), who discovered this pro-inflammatory programmed cell death (PCD) pattern dependent on caspase-1 in Salmonella-infected macrophages (2). Pyroptosis is characterized by the formation of plasma membrane pores and the release of inflammatory contents, accompanied by the occurrence of inflammatory responses (3). The assembly and activation of inflammasome plays a critical role in pyroptosis. It is currently believed that canonical inflammasome activation by caspase-1 as well as noncanonical inflammasome activation through caspase-4, 5, and 11 can both lead to pyroptosis. Importantly, a gasdermin family protein regarded as a executors of pyroptosis, gasdermin D (GSDMD) found in 2015, has been shown to be responsible for pore formation on the plasma membrane (4). Furthermore, when the integrity of the cell membrane is destroyed, a large amount of pro-inflammatory mediators, such as interleukin 1β (IL-1β), interleukin 18 (IL-18), are actively secreted out of the cell through the membrane pores. With the increase of intracellular osmotic pressure, water penetrates into the cell to cause cell swelling, lysis, and induce pyroptosis. After the release of pro-inflammatory mediators and the contents from the cells, the inflammatory cascade is initiated, which can amplify local and systemic inflammation (5).

Pyroptosis not only plays an important role in the body’s immune response and inflammatory response regulation, but also participates in the initiation and/or progression of various diseases including infectious diseases, nervous system diseases, and cardiovascular diseases (CVDs). As an important part of the innate immune system, pyroptosis controls inflammasome-dependent cytokine secretion and contributes to antimicrobial defense. Nevertheless, excessive or inappropriate pyroptosis can be harmful. Several clinical studies showed that inflammasome activation exacerbates immune dysregulation in coronavirus disease 2019 patients, eventually leading to severe diseases (6, 7). Inflammasome and pyroptosis are considered therapeutic targets for coronavirus disease 2019 (8). Pyroptosis is also thought to be involved in neurological diseases such as Parkinson’s disease (9), epilepsy (10) and ischemic stroke (11). A recent bibliometric analysis showed that the study of inflammasome/pyroptosis in the brain is a current and future research hotspot, and it was recommended to study the mechanism of mitochondrial molecules involved in the complex crosstalk of pyroptosis and regulated cell deaths in brain glial cells, which will promote the development of effective treatment strategies (12). In addition, CVD is a chronic condition associated with inflammation. The role of pyroptosis mediated by inflammasome activation in CVDs including atherosclerosis (13), ischemia-reperfusion injury (14), and myocardial infarction (15) has also been gradually confirmed. In general, in-depth study of pyroptosis will help to understand its role in the development and prognosis of related diseases, and provide new ideas for clinical prevention and treatment.

Bibliometrics was first proposed by American bibliographers in 1969. Bibliometrics refers to the discipline that applies mathematical and statistical methods to the study of books and other communication media (16). According to the characteristics of literature database and bibliometrics, bibliometrics can also conduct qualitative and quantitative evaluation of literature research trends. Bibliometrics can not only help scholars quickly grasp the research hotspots and development trends of a specific research field, but also evaluate the distribution of countries/regions, authors, and journals in the research field, laying a foundation for the direction and development of future research (17).

This study aims to explore the hotspots and development trends of pyroptosis in the past 20 years, and draw a map of scientific knowledge with CiteSpace and VOSviewer software, so as to provide new ideas for basic research and clinical prevention and treatment.

## Materials And Methods

### Data Collection

**Figure 1:**
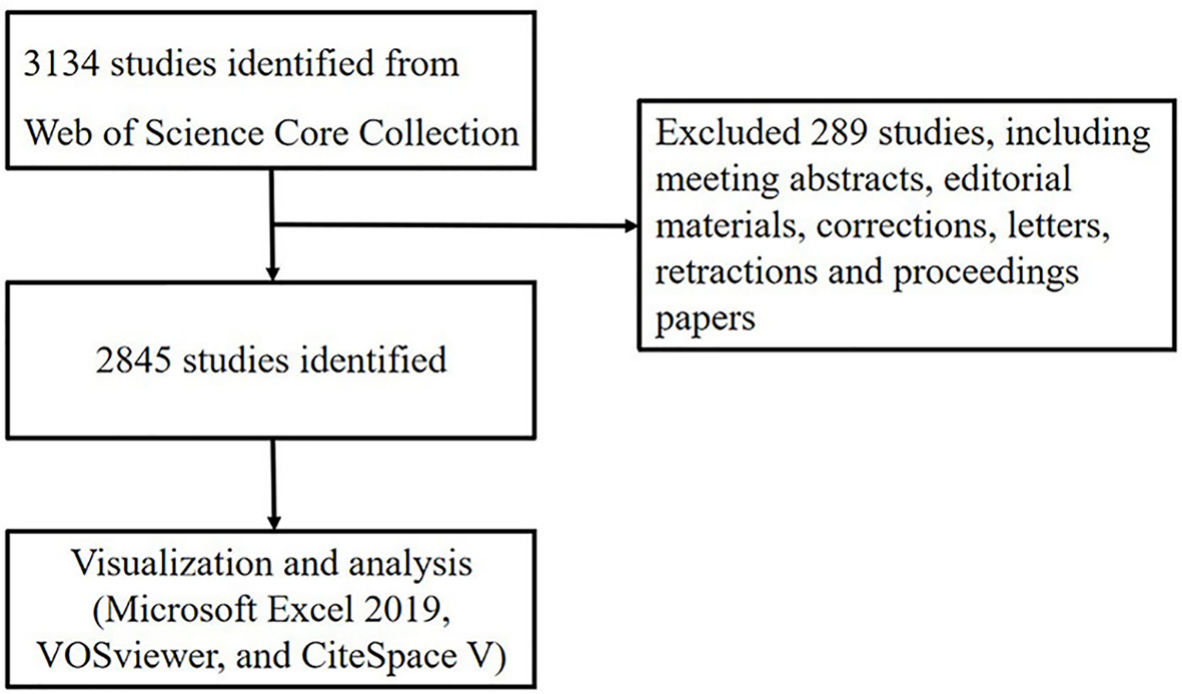
Flowchart of literature selection.

Data were extracted from Web of Science Core Collection and was downloaded within one day on June 14, 2021. The search formula was set to TS =(pyroptosis), and the date of the search were from June 14, 2001, to June 14, 2021. A total of 3,134 articles were retrieved, 289 irrelevant articles including meeting abstracts, editorial materials, corrections, letters, retractions and proceedings paper were excluded. A total of 2845 literatures were exported and the retrieved ones will be exported in the form of all records and references, saved as plain text files and stored in the format of download_txt (**Figure 1**).

### Data Analysis

All valid data were collected in Web of Science Core Collection and imported to Microsoft Excel 2019, VOSviewer and CiteSpace for performing visual analysis.

VOSviewer is a program for building and viewing bibliometric maps. It can be used to build author or journal maps based on collaborative data, or to build keyword maps based on co-occurrence data. The project provides a viewer that allows for a comprehensive and detailed review of bibliometric maps. Different from the commonly used bibliometrics program, VOSviewer pays special attention to the graphic representation of bibliometrics, and it is particularly useful to display large bibliometrics in an easy-to-explain way. The main purpose of developing VOSviewer is to analyze the bibliometric network and build a visual network map, and finally realize a deep and comprehensive understanding of the structure and dynamic development of scientific research (18).

CiteSpace is a citation visualization analysis software that focuses on the analysis of the potential knowledge contained in the scientific literature and is gradually developed in the context of scientometrics and data visualization. The main goal of knowledge domain visualization is to detect and monitor the development of knowledge, which can present the organization, rule and distribution of scientific knowledge through visualization means (19). Knowledge map is a new field driven by information technology, which can intuitively understand the research hotspots and evolution process of various fields in the knowledge system, and predict the development trend of various fields. It is an effective method and means to analyze large-scale data.

We used Microsoft Office Excel 2019 to analyze the trend of the number of articles published in the year, and used CiteSpace and VOSviewer software to analyze the country/region and institutional distribution, author contributions, core journals, keywords and timeline viewer.

## Results

### The Trend of Publication Outputs

**Figure 2:**
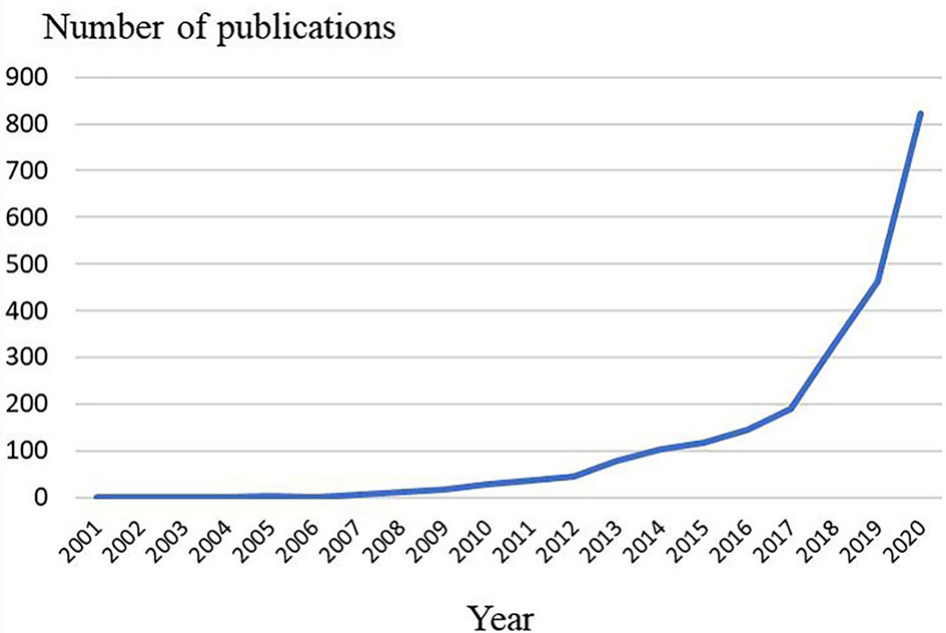
Trends of pyroptosis publications over the past 20 years.

The number of articles published in each period reflects the development trend of research in this field. As is shown in **Figure 2**, the number of articles concerning pyroptosis increased year by year. From 2001 to 2012, publication outputs during this period are extremely low, and the research is still at a standstill. From 2012 to 2017, the amount of literatures have steadily increased, which demonstrates that the field of pyroptosis began to receive attention. From 2017 to 2020, the number of published articles has exploded, and publication outputs have reached 822 in 2020.

### Distribution of Countries/Regions and Institutions

**Figure 3:**
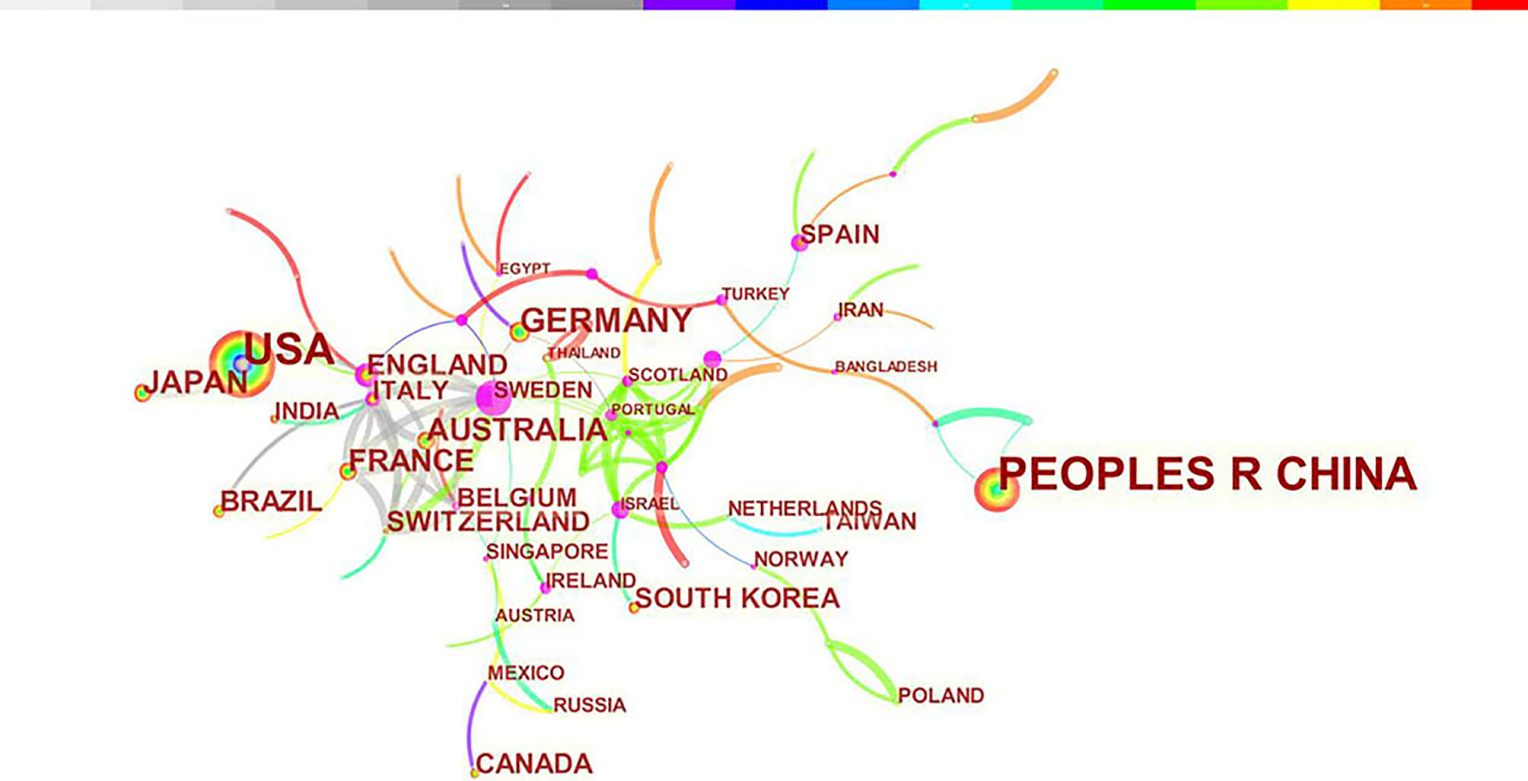
Distribution of publications from different countries/regions.

**Figure 4:**
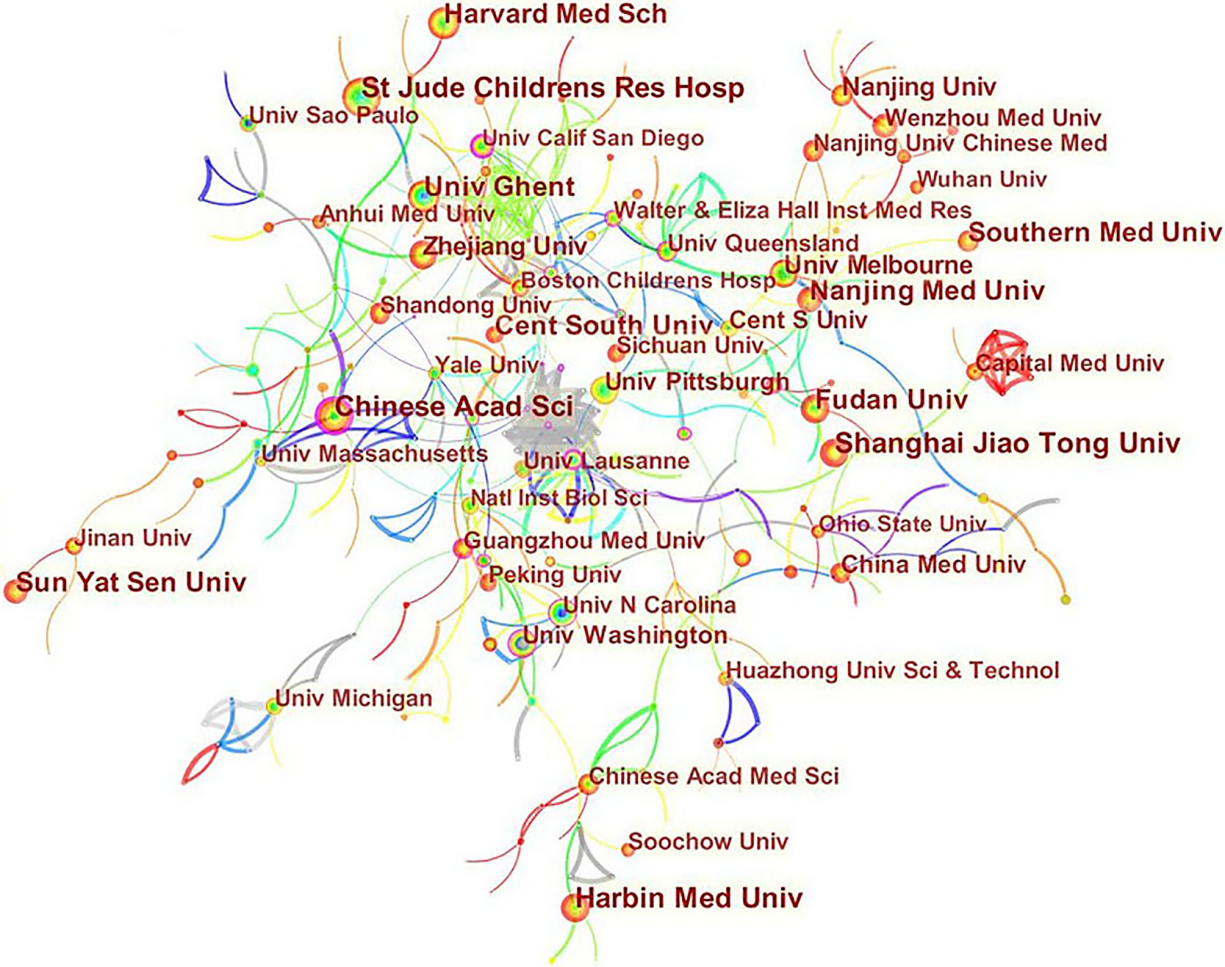
Distribution of publications from different institutions.

A total of 2845 articles were published from 70 different countries and 418 institutions. As can been seen from **Table 1**, the most significant number of publications came from China (1302, 45.76%) and USA (905, 31.81%), which are far more than five times higher than those of other countries. Research institutions with most number of publications are Chinese Acad Sci (67, 2.36%). Among the top 10 institutions, 70% of institutions belong to China. In addition, several countries and institutions, such as England (0.29), Sweden (1.10), Israel (0.40), Denmark (0.40) and Slovenia (0.39) demonstrated a high degree of centrality, as indicated by the purple circles in **Figures 3** and **4**. Each circle in the figure represents a country, and the size of the circle indicates the publication outputs by the country. The lines between the circles denote cooperation between countries, and the wider the lines, the closer the cooperation. A small number of countries and institutions showed active cooperations, such as England, Italy, Australia, France, Belgium and Switzerland, Israel, Portugal and Scotland, Chinese Acad Sci, Univ Massachusetts and Yale Univ. However, most countries and research institutions are scattered and lack stable and intensive cooperation and communication relations.

**Table 1:** Top 10 countries/regions and institutions related to pyroptosis.

Rank	Country	Year	Centrality	Count (%)	Institution	Year	Centrality	Count (%)
1	China	2010	0.00	1302 (45.76%)	Chinese Acad Sci (China)	2010	0.20	67 (2.36%)
2	USA	2005	0.05	905 (31.81%)	St Jude Children’s Res Hosp (USA)	2011	0.01	65 (2.28%)
3	Germany	2010	0.05	163 (5.73%)	Shanghai Jiao Tong Univ (China)	2016	0.00	64 (2.25%)
4	Australia	2009	0.01	125 (4.39%)	Harbin Med Univ (China)	2014	0.01	62 (2.18%)
5	Japan	2007	0.00	117 (4.11%)	Sun Yat Sen Univ (China)	2018	0.01	56 (1.97%)
6	France	2009	0.05	94 (3.30%)	Nanjing Med Univ (China)	2016	0.08	52 (1.83%)
7	England	2007	0.29	86 (3.02%)	Fudan Univ (China)	2016	0.04	51 (1.79%)
8	Brazil	2010	0.00	75 (2.64%)	Ghent Univ (Belgium)	2008	0.04	48 (1.69%)
9	Canada	2007	0.00	68 (2.39%)	Harvard Med Sch (USA)	2016	0.00	48 (1.69%)
10	South Korea	2012	0.00	63 (2.21%)	Southern Med Univ (China)	2018	0.00	47 (1.65%)

### Authors and Co-Cited Authors

**Figure 5:**
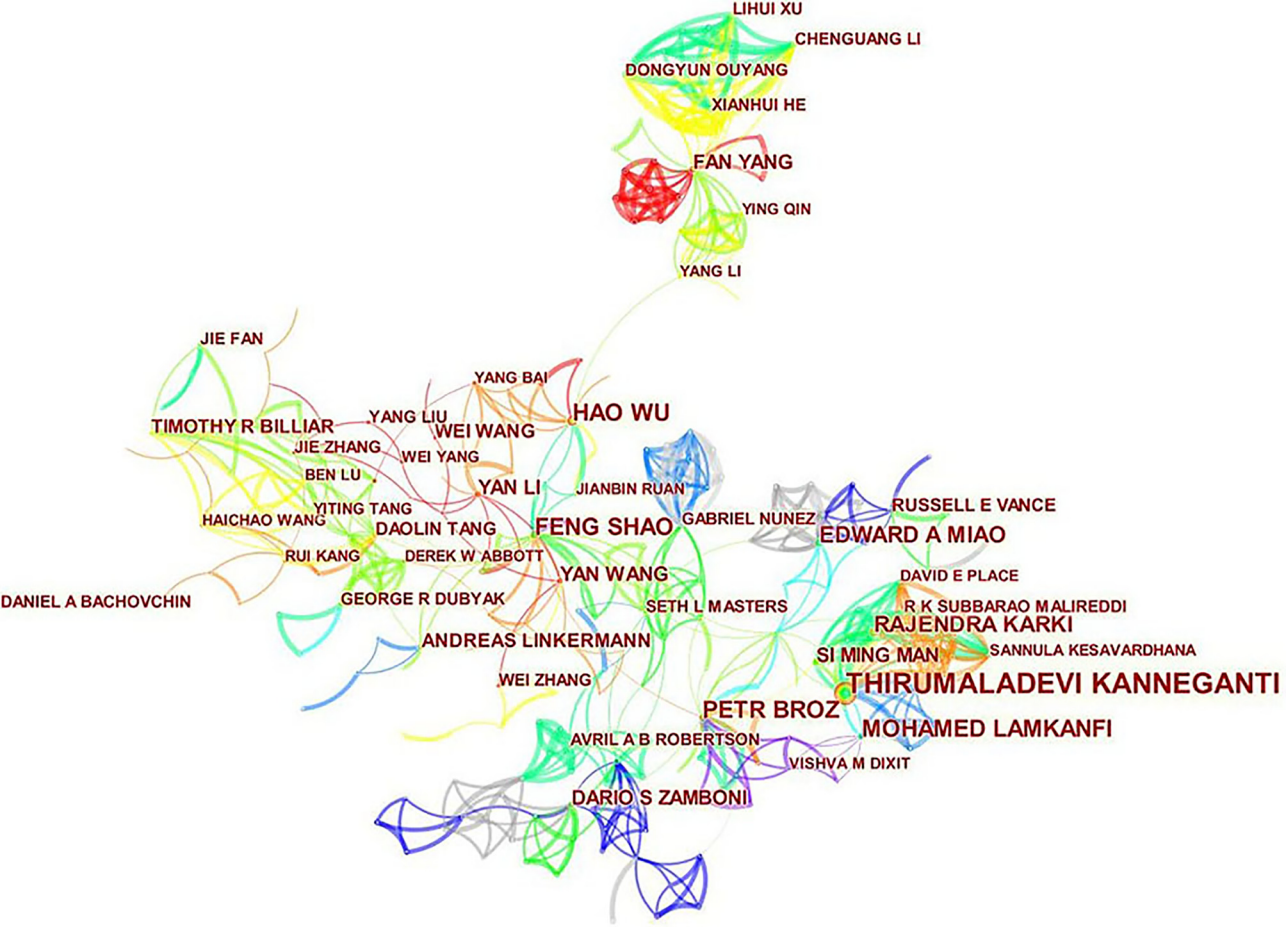
CiteSpace visualization map of authors involved in pyroptosis.

A total of 605 authors were involved in the publication of literature on pyroptosis. As shown in **Table 2**, Thirumaladevi Kanneganti had the highest number of published papers (50, 1.76%). Among the top 10 authors, we can see that Feng Shao (0.18) and Petr Broz (0.13) have high centralities, which shows that these two authors have strong influence on each other’s work as well as works from other groups. Each circle represents an author, the lines between the circles mean the connections between authors, and the connection network of different colors shows the cooperation cluster between different authors. **Figure 5** shows that there is a certain cooperation network between different authors, such as Thirumaladevi Kanneganti, Mohamed Lamkanfi, Rajendra Karki, Si Ming Man, Sannula Kesavardhana, R K Subbarao M Alireddi and David E Place, Timothy R Billiar, Jie Zhang, Ben Lu, Haichao Wang, Rui Kang and Yiting Tang. Co-cited authors are two or more authors who are cited by another or more papers at the same time, and these two or more authors constitute co-cited relationship. Among 1145 co-cited authors, 10 of them have been cited more than 500 times(**Table 2**). Shi JJ (1127) was the most frequently cited author, followed by Kayagaki N (895).

**Table 2:** Top 10 authors and co-cited authors related to pyroptosis.

Rank	Author	Count (%)	Centrality	Co-cited author	Citation	Centrality
1	Thirumaladevi Kanneganti	50 (1.76%)	0.04	Shi JJ	1127	0.01
2	Mohamed Lamkanfi	23 (0.81%)	0.01	Kayagaki N	895	0.02
3	Petr Broz	22 (0.77%)	0.13	Miao Ea	717	0.00
4	Feng Shao	22 (0.77%)	0.18	Lamkanfi M	673	0.00
5	Hao Wu	22 (0.77%)	0.09	Bergsbaken T	649	0.00
6	Edward A Miao	20 (0.70%)	0.02	Fink SL	626	0.01
7	Rajendra Karki	19 (0.67%)	0.02	Broz P	625	0.02
8	Dario S Zamboni	16 (0.56%)	0.04	Martinon F	622	0.01
9	Yan Wang	15 (0.53%)	0.02	Liu X	546	0.01
10	Si Ming Man	14 (0.49%)	0.02	Man SM	539	0.00

### Journals and Co-Cited Academic Journals

We performed a visual analysis of published journals using the VOSviewer software. We found that 2845 articles related to pyroptosis were published in 829 academic journals. The journal *Frontiers in immunology* (90, 3.16%) had the highest number of outputs, followed by *Cell death* & *disease* (73, 2.57%). Among the top 10 academic journals, the highest impact factor (IF) is *Cell death and differentiation* (15.828). Furthermore, it can be seen from **Table 3** that 60% of journals belong to Q1. The influence of journals depends on the number of times they are co-cited, which reflects whether the journal has significant influence in a particular research field. Among the top 10 co-cited academic journals, five journals have been cited more than 5000 times. As shown in **Table 3**, the journals with the highest number of citations is *Nature* (11443), followed by *Proceedings of the national academy of sciences of the United States of America* (6283). According to the 2020 Journal citation reports (JCR), except for *Journal of immunology*, almost all the co-cited journals in the top 10 journals were located in Q1 region.

**Table 3:** Top 10 journals and co-cited journals related to pyroptosis.

Rank	Journal	Count (%)	IF (2020)	JCR	Co-cited journal	Citation	IF (2020)	JCR
1	Frontiers in immunology	90 (3.16%)	7.561	Q2	Nature	11443	49.962	Q1
2	Cell death&disease	73 (2.57%)	8.469	Q1	Proceedings of the national academy of sciences of the United States of America	6283	9.580	Q1
3	Journal of immunology	59 (2.07%)	5.422	Q2	Journal of immunology	6274	5.422	Q2
4	Biochemical and biophysical research communications	44 (1.55%)	3.575	Q2	Cell	5503	41.582	Q1
5	Proceedings of the national academy of sciences of the United States of America	36 (1.27%)	9.580	Q1	Journal of biological chemistry	5503	5.157	Q1
6	Cell death and differentiation	36 (1.27%)	15.828	Q1	Nature immunology	4456	25.606	Q1
7	Plos pathogens	36 (1.27%)	6.823	Q1	Immunity	4236	31.745	Q1
8	International journal of molecular sciences	36 (1.27%)	5.923	Q2	Science	4187	47.728	Q1
9	European journal of immunology	36 (1.27%)	5.532	Q1	Cell death and differentiation	3168	15.828	Q1
10	Scientific reports	36 (1.27%)	4.379	Q1	Plos one	2832	3.240	Q1

IF, impact factor; JCR, Journal Citation Reports.

**Figure 6:**
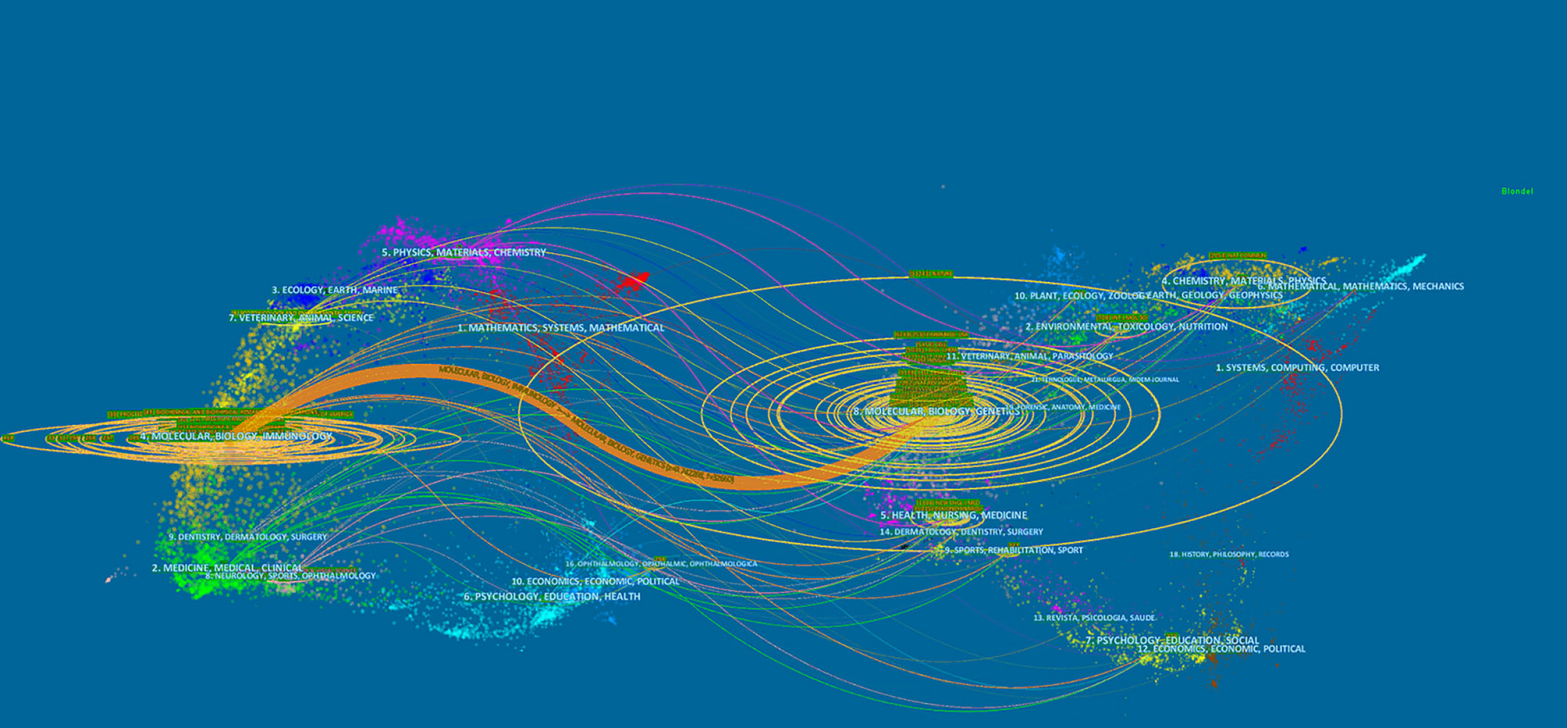
The dual-map overlay of journals on pyroptosis.

The dual-map overlay of journals demonstrated relationship distribution among journals, with citing journals on the left and cited journals on the right, and the colored paths between them suggesting the cited relationships. An orange path in **Figure 6** indicates that the documents published in Molecular/Biology/Genetics journals are often cited by Molecular/Biology/Immunology journals.

### Co-Cited References and References Burst

**Figure 7:**
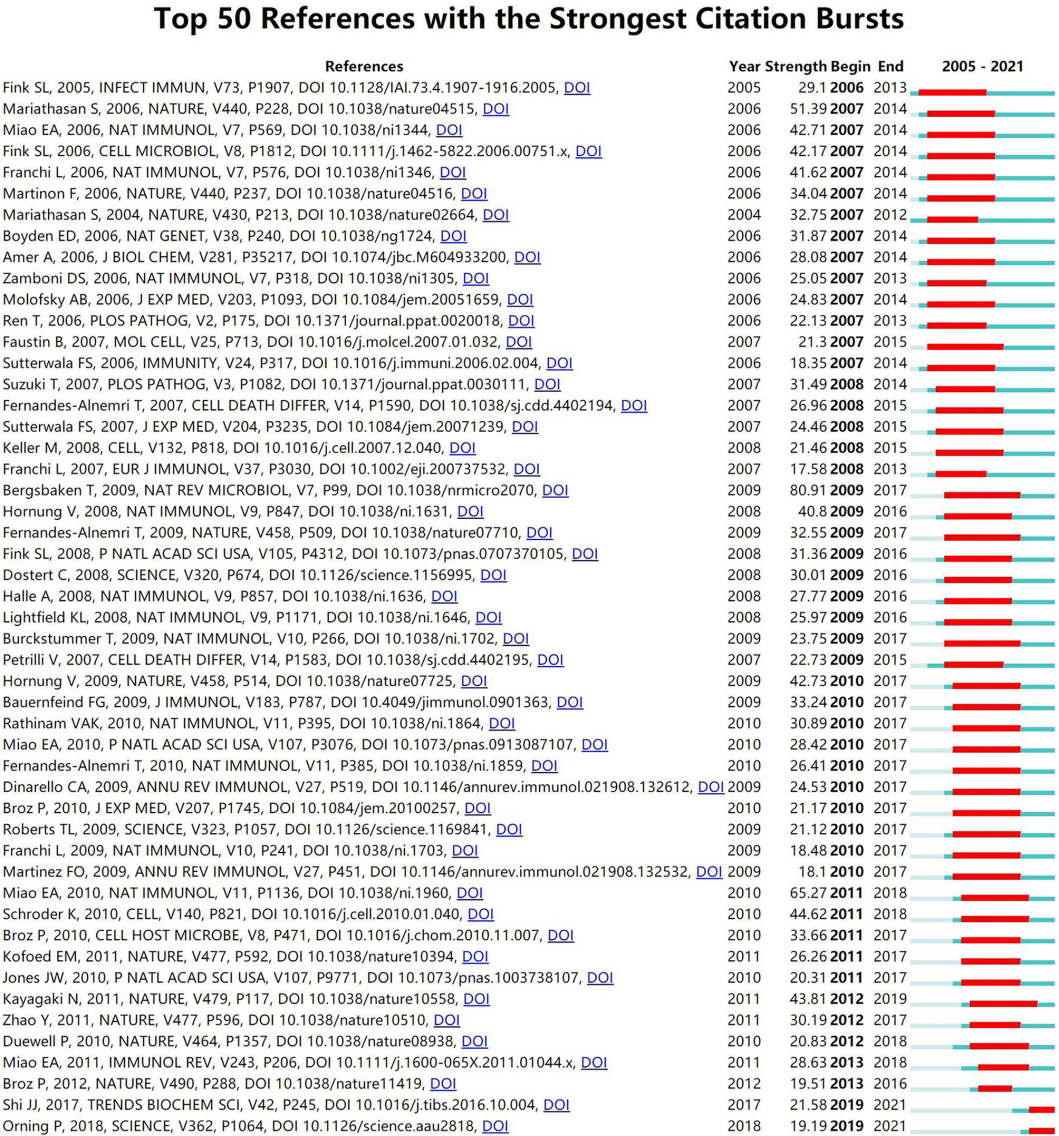
CiteSpace visualization map of top 50 references with the strongest citation bursts involved in pyroptosis.

As a research method to measure the degree of relationship between articles, co-citation refers to that two or more articles are cited by one or more papers at the same time, and the two articles are considered to be a co-citation relationship. Among the 1190 co-cited references retrieved, **Table 4** shows the ten most frequently cited references, of which *Cleavage of GSDMD by inflammatory caspases determines pyroptotic cell death* (4) is the most frequently cited (856). According to references with the strongest citation bursts, **Figure 7** shows that the first burst of co-cited reference began in 2005. The majority of the co-cited references have been frequently cited in recent 10 years, which implies that the research related to pyroptosis may continue to explode in the future.

**Table 4:** Top 10 co-cited references related to pyroptosis.

Rank	Reference	Citation	Year	Centrality	Ref.
1	Cleavage of GSDMD by inflammatory caspases determines pyroptotic cell death	856	2015	0.01	(4)
2	Caspase-11 cleaves gasdermin D for non-canonical inflammasome signaling	631	2015	0.01	(20)
3	Inflammasome-activated gasdermin D causes pyroptosis by forming membrane pores	499	2016	0.00	(3)
4	Pore-forming activity and structural autoinhibition of the gasdermin family	427	2016	0.01	(21)
5	Gasdermin D is an executor of pyroptosis and required for interleukin-1β secretion	389	2015	0.00	(5)
6	Inflammatory caspases are innate immune receptors for intracellular LPS	364	2014	0.01	(22)
7	Pyroptosis: Gasdermin-Mediated Programmed Necrotic Cell Death	352	2017	0.00	(23)
8	Non-canonical inflammasome activation targets caspase-11	346	2011	0.02	(24)
9	Mechanisms and functions of inflammasomes	298	2014	0.00	(25)
10	Chemotherapy drugs induce pyroptosis through caspase-3 cleavage of a gasdermin	293	2017	0.00	(26)

### The Analysis of Hotspots and Frontiers

Keywords are the core of a paper. By analyzing the keywords, we can summarize the study topics in a specific field and explore the hotspots and research directions. According to **Table 5**, excluding for pyroptosis (1459), the keywords appearing at high frequency in this study are nod like receptor family pyrin domain containing 3 (NLRP3) inflammasome (764), apoptosis (687), activation (662), cell death (608), and inflammasome (587). Among these keywords, NLRP3 inflammasome, apoptosis, activation, cell death and inflammasome appeared over 400 times, indicating that these fields were the hot topics in the study of pyroptosis.

**Table 5:** Top 10 keywords related to pyroptosis.

Rank	Keywords	Count	Centrality	Rank	Keywords	Count	Centrality
1	pyroptosis	1459	0.02	11	expression	302	0.02
2	NLRP3 inflammasome	764	0.01	12	NF-κB	259	0.04
3	apoptosis	687	0.02	13	autophagy	230	0.03
4	activation	662	0.02	14	oxidative stress	217	0.03
5	cell death	608	0.01	15	macrophage	206	0.03
6	inflammasome	587	0.01	16	protein	191	0.03
7	GSDMD	455	0.01	17	necroptosis	188	0.01
8	mechanism	399	0.02	18	death	183	0.01
9	caspase-1	395	0.02	19	innate immunity	180	0.01
10	inflammation	355	0.03	20	receptor	171	0.06

**Figure 8:**
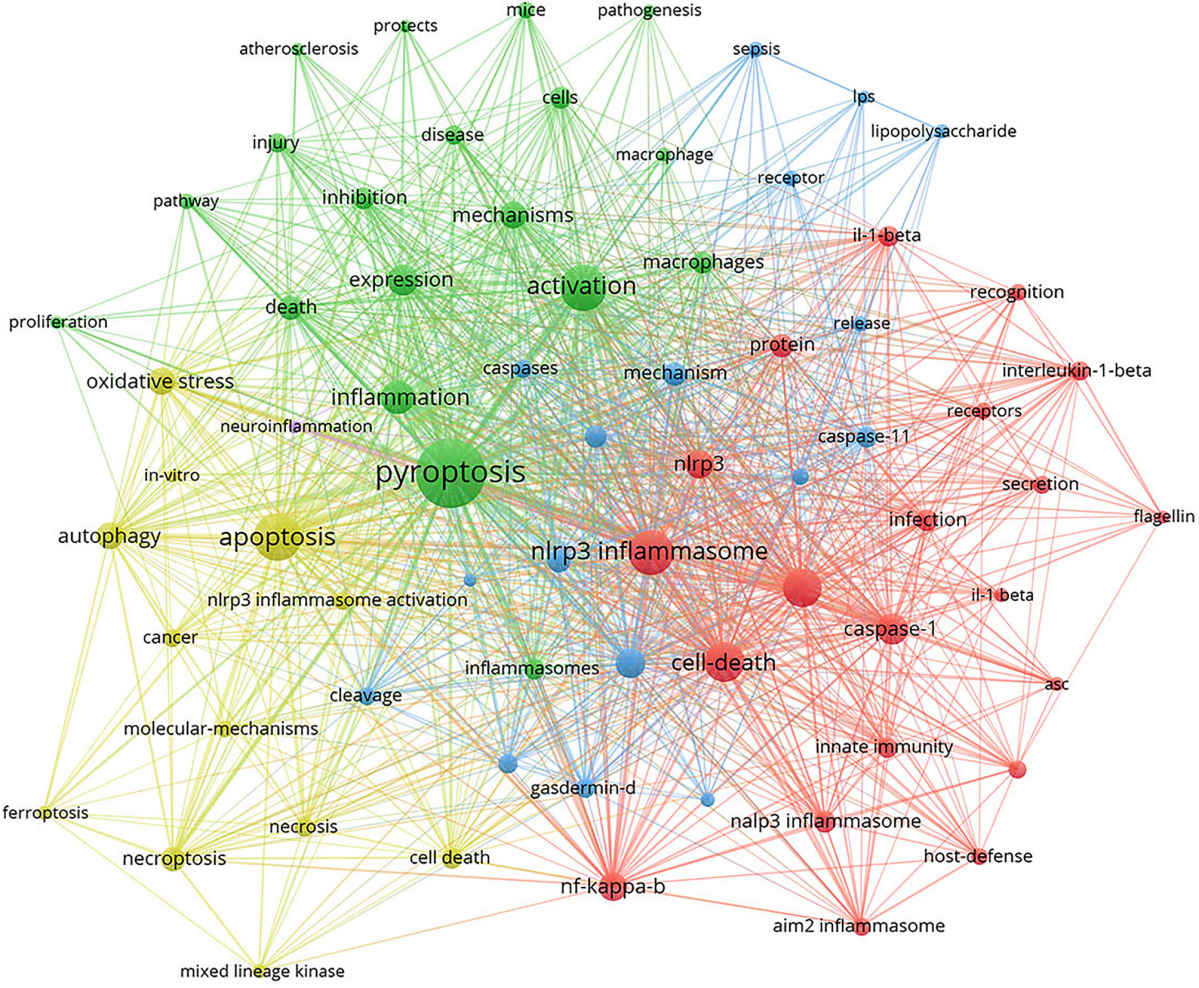
CiteSpace visualization map of keywords clustering analysis related to pyroptosis.

Based on the analysis of keyword co-occurrence, the network map is clustered, which reflects the basic knowledge structure of related research fields. We use VOSviewer software to cluster keywords from literatures. Circles and labels form a unit, and units of different colors are made up for different clusters. As shown in **Figure 8**, we can see the clusters of red, green, blue and yellow, which respectively represent four different research directions. The main keywords of green cluster are pyroptosis, activation, mechanisms and pathway. The keywords of red cluster are cell death, inflammasome, caspase-1 and infection. While the keywords of blue cluster include GSDMD, cleavage and expression, and of yellow cluster mainly include apoptosis, autophagy, necrosis and ferroptosis.

**Figure 9:**
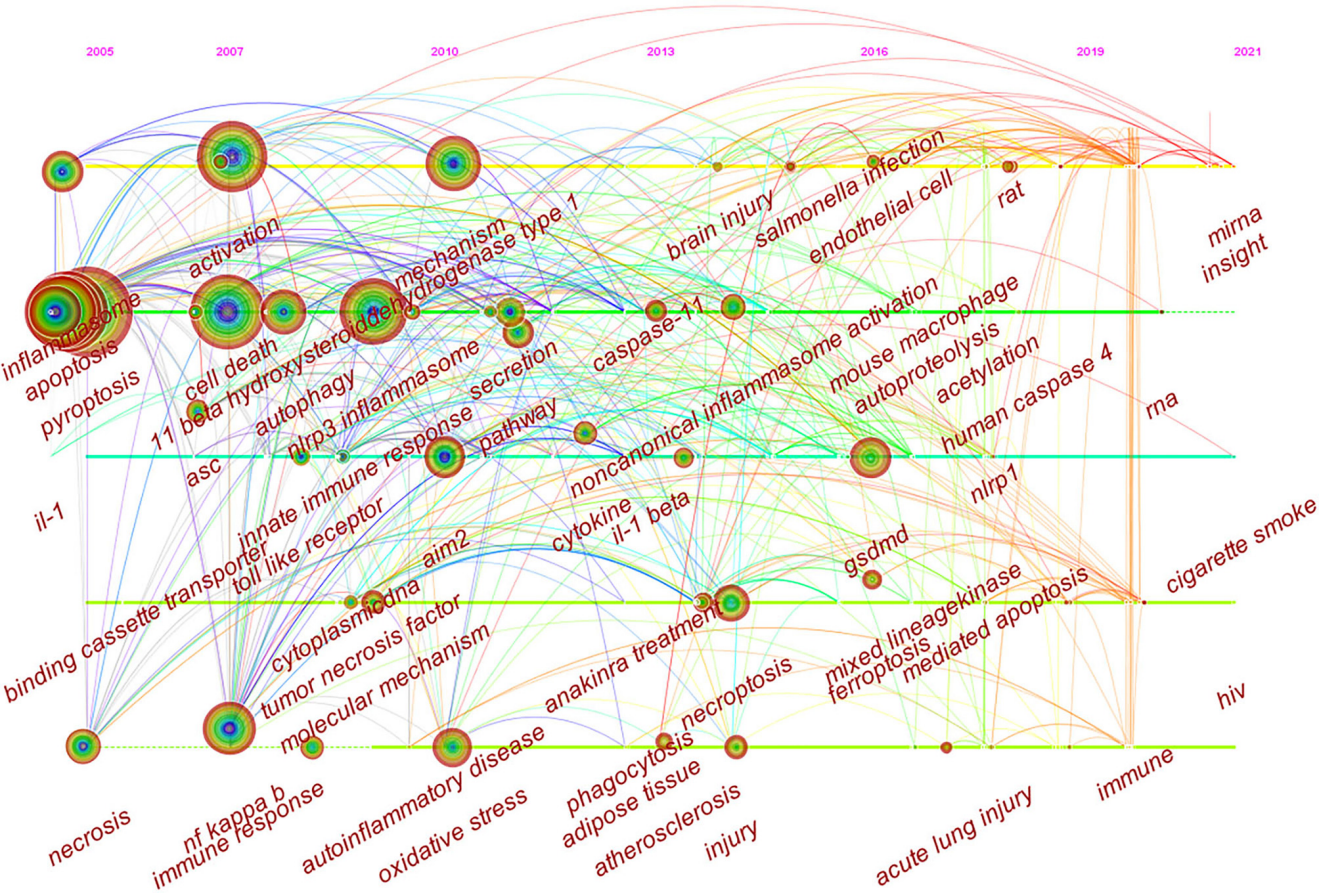
CiteSpace visualization map of timeline viewer related to pyroptosis.

Timeline viewer is based on the interaction and mutation relationship between keywords in a certain field, which is helpful for exploring the evolution track and stage characteristics of the research field. **Figure 9** is the timeline viewer of focal death drawn based on CiteSpace software in this study, which visually shows the phased hotspots and development directions of the research on pyroptosis from the time dimension. From 2005 to 2011, the research mainly focuses on the functional analysis of inflammasomes and related mechanisms, and the main keywords are activation, cell death, ASC, NLRP3 inflammasome, innate immune response, toll like receptor, AIM2, tumor necrosis factor, inhibition and oxidative stress. From 2011 to 2021, the research is mainly based on the downstream pathway mechanism of pyroptosis, and the main keywords are caspase-11, GSDMD, human caspase-4, and mixed lineagekinase. It is worth noting that in the past 20 years, the study of pyroptosis has been closely related to various diseases (**Figures 8** and **9**), such as autoinflammatory diseases, atherosclerosis, ischemia-reperfusion injury, stroke, acute lung injury and brain injury.

## Discussion

### General Information

The annual number and trend of literatures can reflect the development speed and research progress of this study, and can also indicate the concentration of research in this field. It can be seen from **Figure 2** that the overall number of publications is on the rise. Specifically, from 2001 to 2012, the relatively small amount of literature in this period indicates that the study of pyroptosis is in its infancy. From 2012 to 2017, the amount of literature showed a steady growth. During this period, the protein GSDMD, which is considered as the executor of pyroptosis, was proposed (4). GSDMD is a common substrate of inflammatory caspases, which not only clearly illustrates the molecular basis of pyroptosis, but also redefines pyroptosis as gasdermin-mediated programmed cell necrosis, opening up a new path for subsequent studies on programmed cell necrosis and innate immunity. From 2017 to 2020, research related to pyroptosis has exploded and the number of publications has reached 822 in 2020. It can be seen that the research related to pyroptosis is in the hot study direction in recent years, and has an excellent development trend in the future.

According to the distribution of countries/regions and institutions in **Table 1**, we can see that the country with the highest number of publications is China (1302, 45.76%), followed by the USA (905, 31.81%), which together account for 77.57% of the total. This indicates that China and the USA are the leading countries on pyroptosis research. Centrality is an index to measure the importance of a node in a network. It is mainly used to measure the value of the bridge function of the node in the entire network structure. Generally, nodes greater than 0.1 are considered as relatively important nodes. Among the top 10 countries in **Table 1**, England has the highest centrality (0.29), which means it plays a key role as a bridge in the worldwide network of state cooperation. Among the top ten research institutions, seven are from China and two are from the USA, with the Chinese Academy of Sciences (0.20) having the highest impact. Nevertheless, from **Figures 3** and **4**, the distribution of individual countries and institutions are scattered, and the main research subjects, such as China and USA, the Chinese Academy of Sciences and St Jude Children’s Research Hospital do not form a network, indicating a lack of academic exchange between countries as well as research institutions. This situation hinders the development of this research field. Therefore, it is strongly suggested that the US and China and research institutions from other countries should remove academic barriers, cooperate, and communicate to promote the research and development of pyroptosis.

From the perspective of authors and co-cited authors, Thirumaladevi Kanneganti (50, 1.76%) is the author with the highest number of publications, followed by Mohamed Lamkanfi (23, 0.81%), Petr Broz (22, 0.77%), Feng Shao (22, 0.77%), and Hao Wu (22, 0.77%). It is noteworthy that Feng Shao (0.18) exerts the significant greatest publication impact and have made the most outstanding contributions in the field of pyroptosis. Feng Shao, a biochemist, was elected as an academician of Chinese Academy of Sciences in 2015, mainly engaged in the molecular mechanism of pathogenic bacteria infection and innate immune defense of hosts. In 2015, Feng Shao and his colleagues identified that GSDMD is a common substrate for caspase-1 and caspase-4/5/11, and is a key effector molecule for mediating inflammatory cell death (4). This discovery not only reveals the mechanism and function of GSDMD, which may provide a new way for the treatment of inflammation-caspase-related autoimmune state and septic shock, but also redefines the concept that pyroptosis is programmed necrosis mediated by gasdermin. Recently, Feng Shao and Zhibo Liu revealed the anti-tumor immune function of pyroptosis by constructing biorthogonal system, and found that gasdermin agonist could improve the efficacy of cancer immunotherapy. The effect on immune-dependent anticancer chemotherapy drugs may be due to the activation of gasdermin (27). This indicates that the research on the relationship between pyroptosis and diseases has gradually become a hot topic. As for co-cited authors, the articles of 8 authors were cited more than 500 times, with Shi JJ (1127) having the highest number of citations, followed by Kayagaki N (895), and Miao Ea (717).

According to the journals and co-cited journals in **Table 3**, the journals with the most articles about pyroptosis were *Frontiers in immunology* (90, 3.16%), followed by *Cell death* & *disease* (73, 2.57%), *Journal of immunology* (59, 2.07%). It can be seen that the research on the mechanism of pyroptosis in immunity and diseases is a hot topic at present and also a development trend in the future. As for co-cited journals, *Nature* (11443) is a journal with highest citations, followed by *Proceedings of the national academy of sciences of the United States of America* (6283), *Journal of immunology* (6274), *Cell* (5503), and J*ournal of biological chemistry* (5062). As is shown in **Table 3**, five journals cited more than 5000 times. Among the top ten journals, nine belong to Q1. The analysis of the distribution of literature sources is helpful to find the core journals published in the research regarding pyroptosis. It can be seen that the cited literatures are all from high-impact journals, indicating that research on pyroptosis is highly valued in the global academic field.

Among top 10 co-cited references related to pyroptosis, the top five co-cited references are all about gasdermin proteins, especially GSDMD. According to top 50 references with the strongest citation bursts, it can be seen that the most cited reference in recent years is *Pyroptosis: Gasdermin-Mediated Programmed Necrotic Cell Death*, which indicates that the gasdermin family suggests a new area of research on pyroptosis function in immunity and disease. Nevertheless, the function of many gasdermins and their mechanism of activation remain to be proved.

### The Hotspots and Frontiers

Keywords are the research themes and core contents of the literature. Based on keyword co-occurrence analysis, it is possible to understand the distribution and development of different research hotspots in a certain field. It can be seen from **Table 5** that the keywords with high occurrence frequency are pyroptosis (1459), NLRP3 inflammasome (746), apoptosis (687), activation (662), cell death (608), inflammasome (587), and GSDMD (455). Cluster analysis was conducted on the basis of keywords, and finally four colors clusters were formed. Then, according to the timeline viewer analysis of clustering, we determine the research hotspots and development frontiers in the field of pyroptosis. The main contents are as follows:

#### The Pathway Mechanism of Pyroptosis

Pyroptosis is mainly mediated by inflammasomes to activate various caspases, including caspase-1. Inflammatory caspases can directly shear and activate GSDMD, mediate the formation of plasma membrane pores, and cause cell death. Meanwhile, with the release of a large number of pro-inflammatory factors such as IL-1β and IL-18, inflammatory cascade waterfalls are triggered (28, 29).

Inflammasomes, first proposed by the Tschopp team in 2002, play an important role in innate immunity (30). Inflammasomes are polyprotein complexes that consist of pattern recognition receptors(PRRs), the adapter protein apoptosis-associated speck-like protein containing caspase recruitment domain(ASC), and the effector molecule pro-caspase (31). PRRs can detect pathogen-associated molecular patterns(PAMPs) of unique microbial structures such as bacteria, or host-derived risk/damage associated molecular patterns(DAMPs) including high mobility group protein 1 (HMGB1), heat shock protein (HSP), S100 protein, ATP, and uric acid crystals to recruit and activate inflammatory caspases (32). To date, the inflammasomes associated with pyroptosis that have been studied extensively include NLRP1, NLRP3, NLRC4 (also known as IPAF) inflammasomes in the NLR family and AIM2 inflammasomes, of which NLRP3 is the inflammasome that has been studied most concretely and its mechanism is relatively well understood. NLRP3 protein has three basic domains, including the C-terminal leucine-rich repeat(LRR) domain, which has the function of ligand recognition; the nucleotide-binding and oligomerization domain(NACHT) in the central region, which is important for the oligomerization and activation of the receptor itself; and the N-terminal pyrin domain(PYD), which binds to ASC through PYD-PYD. ASC has the PYD and caspase recruitment domain (CARD), which connects with the upstream NLRP3 protein through PYD and the downstream pro-caspase-1 through CARD (33–35). Other subtypes in the NLR family have similar domains. A variety of pathogens and intracellular and extracellular stimuli are recognized by LRR. The NLRP3 protein structure changes, exposing the NATCH domain, and then polymerizes to form a highly ordered NLRP3 protein oligomer, and recruits ASC through the PYD-PYD structure. ASC recruits pro-caspase-1 through the Card-Card domain to complete the assembly of inflammasomes. The assembled NLRP3 inflammasomes had the function of self-activation, leading to the hydrolysis of pro-caspase-1 to generate active caspase-1, and further promoting the maturation of IL-1β and IL-18 (36–41). However, there are also inflammasomes such as (NLRP1 and NLRC4) which do not need the adaptor protein ASC and whose receptor themself have CARDs that can be directly connected to pro-caspase-1 (42, 43). Additionally, AIM2 inflammasome is composed of AIM2, connective protein ASC and effector protein caspase-1. The AIM2 protein has a C-terminal HIN200 domain and an N-terminal PYD. After assembly, the AIM2 inflammasome can promote caspase-1 activation and the maturation of IL-1β and IL-18 (44, 45). According to our analysis, the mechanism of the relationship between other types of inflammasomes such as NLRP1 and NLRC4 and pyroptosis has also gradually attracted attention.

Caspase is a family of proteases with many members, and pyroptosis is mainly mediated by inflammatory caspase-1 (46–48). Recent studies have also shown that pyroptosis can also be mediated by human caspase-4 (49), caspase-5 (50) or mouse caspase-11 (51). Pro-caspase-1 was hydrolyzed by activated inflammasome to form active P10/P20 tetramer. Caspase-1, also known as interleukin-1β converting enzyme, can promote the cleavage and maturation of pro-IL-1β and pro-IL-18 to produce active IL-1β and IL-18, which are important pro-inflammatory cytokines involved in multitude of human diseases (52). In addition, caspase-1 can also directly shear and activate GSDMD protein, mediate the formation of plasma membrane pores and induce pyroptosis. This is the classic way of pyroptosis. While non-classical pathways are mainly mediated by human homologous genes caspase-4, caspase-5 or mouse caspase-11, which can be activated by various Gram-negative bacterial infections. Lipopolysaccharide(LPS) is the PAMP that promotes Caspase-11 activation. LPS in the cytoplasm can directly bind to and activate caspase-11 independently of toll-like receptor 4(TLR4). Caspase-11 can also trigger the activation of classical pathways and mature activation of pro-IL-1β through pyroptosis, which leads to the death of infected cells (20, 24, 53–55). Although there is no caspase-11 in human body, its homologous caspase-4 and caspase-5 have the same function and activation method as caspase-11. To date, non-classical pathways of pyroptosis have attracted more attention and become hot research directions (56–58).

In 2015, Feng Shao et al. identified GSDMD by genome-wide clustered regularly interspaced palindromic repeat (CRISPR)-Cas9 nuclease screens of caspase-11- and caspase-1-mediated pyroptosis in mouse bone marrow macrophages, and proposed that GSDMD is a central mediator of caspase-1 and caspase-4/5/11 downstream causing pyroptosis and is essential for IL-1β secretion (4). GSDMD is a member of the gasdermin protein family that also includes gasdermin A, B, C, D, E (also known as DFNA5) and DFNB59 (59, 61). GSDMD is generally found in the cytoplasm and is in an auto-inhibited state at steady state. When activated inflammatory caspases cleave the GSDMD protein, an N-terminal P30 fragment (GSDMD-NT) and a hydrophilic C-terminal P20 fragment (GSDMD-C) are formed. GSDMD-NT can specifically bind to the lipid bilayer of the cell membrane, forming small pores of 10-15nm in diameter, also known as gasdermin pores, which disrupt the cell membrane integrity, a large number of pro-inflammatory mediators IL-1β and IL-18 are actively secreted outside the cell, water enters the cell, the cell swells, lyses, and induces pyroptosis (21, 62). GSDMD-C also has an important role in inhibiting the cytotoxicity of GSDMD-N, promoting the water solubility of GSDMD-NT, and exerting a structural self-inhibitory effect when it binds to GSDMD-NT (49). Elucidating the structure of GSDMD pores, pore-forming mechanism and the means of inflammatory factor secretion have always been difficult points in the study of pyroptosis. Recently, Hao Wu’s team demonstrated the high-resolution structure of GSDMD pores, proposed the pore-forming mechanism of GSDMD, and revealed the molecular mechanism of IL -1 release through structural analysis and functional experiments. GSDMD contains 31 to 34 subunits, with the highest pore resolution of 33 subunits. Its transmembrane domain is a large barreled structure surrounded by 132 β strands, called the β-barrel. Next to the β-barrel there was a layer of globular domain on the cytosolic side. In addition, they also showed GSDMD prepore, which is globular and lacked β-barrel, and its conformation was quite different from that of GSDMD pores. The biological significance of GSDMD prepore is not clear, but the authors suggest that it may be an intermediate state during the pore assembly of GSDMD. In addition to the charge interaction between the α1 helix of GSDMD and lipid molecules on the cell membrane, GSDMD also has two charge-rich regions that are important for GSDMD to bind to the cell membrane. Surprisingly, the closest region of GSDMD prepore to the cell membrane is a hydrophobic loop structure called β1-β2 loop, which affects the pore formation efficiency of GSDMD. There was a large accumulation of negative charges in the pores of GSDMD, especially in the area near the cytoplasm. Interestingly, the IL-1 precursor surface charge was negative, while the mature IL-1 surface charge was positive. Based on these analyses, the authors proposed and validated the importance of charge for IL-1 release, which is another major progress related to pyroptosis (63). Petr Broz et al. found that when the cells execute pyroptosis, the calcium ion influx through the GSDMD hole could be used as a signal to start the membrane repair, and recruited the endosomal sorting complexes required for transport (ESCRT) machinery to the damaged membrane area. After activation of typical or atypical inflammasomes, inhibition of ESCRT-III can significantly increase the rate of pyroptosis. This study found a remedy mechanism in the process of endogenous pyroptosis, which provides a new idea for the treatment of inflammatory diseases (64). Recently, Wandel and colleagues found that guanylate-binding proteins(GBPs) assembled on the surfaces of Salmonella typhimurium and Shigella flexneri to form the multivalent signaling platform required for caspase-4 activation in interferon-gamma-stimulated cells. The assembly of platform, the recruitment of caspase-4 and the activation of caspase-4 are controlled by specific GBPs. In response to invading cytoplasmic bacteria, activation of caspase-4 *via* the GBP platform is critical for inducing GSDMD-dependent pyroptosis and IL-18 processing, thereby destroying intracellular bacterial replication sites and alerting adjacent cells (65). This achievement has been an important finding in recent years, providing new ideas for the various activation mechanisms of the gasdermin family. Additionally, studies reported that other types of gasdermin proteins can also induce pyroptosis through other pathways. For example, Hu et al. have found that tumor necrosis factor α+ cycloheximide and navitoclax-induced cancer cell pyroptosis through a BAK/BAX-caspase-3-GSDME signaling pathway. GSDME knockdown inhibited the pyroptosis, indicating the essential role of GSDME in this process (66). Our study also suggests that other types of gasdermin proteins will receive increasing attention in the future.

#### Comparison and Crosstalk of Pyroptosis With Other Types of Cell Death

Cell death can be divided into two major categories: PCD and non-programmed cell death. PCD includes apoptosis (58–60), autophagy (67), pyroptosis, necroptosis (68), and ferroptosis (69), while non-programmed cell death includes necrosis.

PCD plays a crucial role in biological development, normal physiological activities and various diseases. Different types of PCD do not play a single role, and there are several evidences have shown that different PCD pathways are interconnected at multiple levels. A study has demonstrated that pyroptosis stimuli can activate apoptosis through a variety of mechanisms. On the contrary, apoptotic stimuli can avoid or even inactivate pyroptosis. Taabazuing and colleagues found that small-molecule inhibitors of serine peptidases DPP8 and DPP9 (DPP8/9) selectively induced caspase-1-dependent pyroptosis in monocytes and macrophages. However, in the absence of GSDMD, the active caspase-1 induced apoptosis by activating caspase-3/7, confirming that GSDMD was the only caspase-1 substrate that induced pyroptosis in cells. In addition, they also confirmed that caspase-3/7 and inflammatory caspase have different cleavage sites for GSDMD, and caspase3/7 can cleave to inactivate GSDMD, specifically blocking pyroptosis. Thus revealing the complex interactions between the pyroptosis and apoptosis pathways in innate immune cells (70). Another study also supports the existence of crosstalk between pyroptosis and apoptosis. Orning et al. identified that caspase-8-mediated cell death triggered by pathogens is involved in the inflammatory pathway of GSDMD. It was confirmed that caspase-8 activated GSDMD by hydrolysis after Yersinia infected macrophages, which triggered the process of pyroptosis (71). Sarhan et al. also supported that in the process of transforming growth factor beta-activated kinase 1 inhibition, the activation of caspase-8 led to the cleavage of GSDMD in mouse macrophages, leading to pyroptosis. When the absence of GSDMD delayed membrane rupture, the cell death morphology turned to apoptosis (72). Another recent study suggested that the activity of caspase-8 was the key switch between apoptosis, necroptosis and pyroptosis, and determined the fate of cells by inducing crosstalk between them (73). Enzyme-inactivated caspase-8 could not induce apoptosis, but it mainly induced lysis and death through necroptosis mechanism. Nevertheless, when necroptosis was blocked, inactive caspase-8 induced pyroptosis, leading to severe inflammatory reaction (74). These studies further demonstrated that there is no strict boundary between apoptotic caspase and inflammatory caspase in the regulation of downstream molecules. The pathogen and its secretion system components affect different signaling proteins simultaneously and may involve multiple pathways of cell death pathway.

Notably, crosstalk between different types of PCD has crucial implications for clinical applications as well. A recent study showed that GSDME can be cleaved by caspase-3, and the cleavage site and result are extremely similar to caspase-1 cleavage of GSDMD, and it forms and releases an N-terminal domain with pore-forming activity to induce cell pyroptosis. Additionally, caspase-3 also plays an important role in apoptosis. Due to epigenetic silencing mediated by DNA methylation, GSDME is not expressed in cancer cells but is highly expressed in many normal tissues. Therefore, after caspase-3 is activated by treatment with chemotherapy drugs, these normal cells will undergo GSDME activation and die through pyroptosis. Since cancer cells do not express GSDME, caspase-3 activation induces cancer cells to die in an apoptotic manner. However, GSDME^-/-^ mice can be protected from a variety of side effects such as tissue damage and body weight loss caused by chemotherapy drugs, which indicates that traditional chemotherapy drugs have great toxic and side effects, which may be due to the pyroptosis of normal tissues caused by the drugs (26). In general, understanding the crosstalk between different types of PCDs will be helpful for clinical diagnosis and treatment.

#### Role of Pyroptosis in Diseases

When stimulated by exogenous pathogens or host-derived hazard signals, pyroptosis is responsible for cell lysis and the release of various pro-inflammatory cytokines, such as IL-1β and IL-18. Normally, moderate pyroptosis is conducive to the timely removal of infected cells. Nevertheless, excessive activation of pyroptosis leads to a large number of cell deaths and inflammatory cascade reactions, seriously damaging tissues and organs, and even leading to organ failure, which is closely related to the pathogenesis of certain diseases (75–79). A large number of studies have shown that pyroptosis is widely involved in the occurrence and progression of infectious diseases (80), metabolic diseases (81), tumors (82), nervous system diseases (83) and cardiovascular diseases (84). To date, pyroptosis is a highly regulated cell death process, and its regulation by drugs or other treatments may have a protective effect in various diseases (**Table 6**).

**Table 6:** The role of pyroptosis in different diseases.

Disease	Promote (+)/Suppress (–) diseases	Model	Agent	Cell	Mechanism	Ref.
Epilepsy	–	rats	Chaihu-Longgu-Muli decoction	hippocampal neurons	reduce the expression of NLRP3, caspase-1 and IL-1β	(10)
Atherosclerosis	–	apoE^-/-^ mice	melatonin	endothelial cells	inhibit pyroptosis through MEG3/miR-223/NLRP3 axis; reduce the expression of GSDMD, IL-1β, IL-18, NF-κB	(85)
Hepatocellular carcinoma	–	transgenic mice	sorafenib	macrophages	up-regulate caspase-1	(86)
Alcoholic liver disease	+	C57BL/6 mice	ethanol	hepatocytes	facilitate NLRP3 inflammasome activation	(87)
Ischemic heart disease	–	H9c2 cells	liraglutide	H9c2 cells	inhibited the activation of NLRP3 inflammasome *via* SIRT1; suppressed TNF-α and hypoxia-induced pyroptosis	(88)
Atherosclerosis	–	cells	microRNA-30c-5p	human aortic endothelial cells	weaken the effect of caspase-1, IL-18, and IL-1β at protein level and transcriptional level	(89)
Food-borne gastroenteritis	+	STAT1^-/-^ mice	norovirus	macrophages	activate NLRP3 inflammasome	(90)
Infectious and inflammatory diseases	–	wild-type mice	type I interferons	bone marrow -derived macrophages	induced the expression of caspase-11 and GSDMD; activate NLRP3 inflammasome	(91)
Colon cancer	–	cells	liver X receptor β agonists	colon cancer cells	activate NLRP3 inflammasome and caspase-1 through the P2RX7 pathway	(92)
Diabetic nephropathy	+	rats	Long noncoding RNA MALAT1	HK-2 human proximal tubular epithelial cells	activate NLRP3 inflammasome, caspase-1, IL-1β	(93)

Pyroptosis is a double-edged sword. This contradiction is particularly significant in cancer and its treatment. Tumors can use a variety of strategies to avoid or limit the pathways of cell death. On the other hand, tumors can be killed in different ways (81, 82). This complex mechanism poses a challenge to clinical diagnosis and treatment. To date, a large number of studies have begun to focus on targeted therapy strategies centered on molecules in various links of the pyroptosis, such as NLRP3 inflammasome, caspase-1, and GSDMD. Although the role of the pyroptosis effector protein GSDMD has gradually emerged in recent years, many researchers have begun to look for inhibitors of GSDMD, hoping to block the process of pyroptosis (94, 95). In 2018, Rathkey et al. found necrosulfonamide, a chemical blocker of GSDMD, in a model of sepsis, likely through alkylating a key cysteine residue (Cys191in human or Cys192 in mouse), which destroyed the function of GSDMD-NT domain and pore formation (96). A recent study also showed that dithiamine, an FDA-approved drug for the treatment of alcohol addiction, inhibited the formation of membrane pores by covalently modifying Cys191/Cys192 in GSDMD (97). According to relevant studies (98, 99), Cys191/Cys 192 has been found to be the most effective site for the inhibition of GSDMD, followed by the inhibition of unknown sites of GSDMD-NT, and regulation of the cleavage between caspase and GSDMD. Additionally, several NLRP3 inflammasome inhibitors such as colchicine, OLT1177 and MCC950 have also been identified to inhibit the activity of ATPase and prevent the assembly and activation of NLRP3 inflammasome. Caspase-1 inhibitors, including VX-765, are also used in the treatment of CVDs and infectious diseases (100). Although VX-765 has a high tolerance for effectively reducing inflammatory reaction, its clinical effect is not optimistic, and clinical data show that its efficacy is lower than expected. In general, although a great deal of evidence shows the effectiveness of pyroptosis inhibitors in animal experiments, there is still a lack of large-scale clinical data to prove the safe dose and clinical efficacy of pyroptosis inhibitors.

## Limitation

CiteSpace and VOSviewer software can not completely replace system retrieval, and there are still some limitations to be solved. First of all, the literature we obtained was from 2001 to 2021. However, with the continuous updating of the literature in WoSCC, the retrieval results of this study are somewhat different from the actual number of included literatures. Secondly, this study includes both articles and reviews, and the uneven quality of the collected literatures may reduce the credibility of the map analysis. Finally, several core keywords in the paper were not included entirely in the analysis, and the results may be affected by incomplete keyword extraction. However, the visualized analysis based on literature undoubtedly lays a foundation for scholars to quickly understand the research subjects, research hotspots and development trends in the field of pyroptosis.

## Conclusion

The research on the mechanism of pyroptosis, the crosstalk between different types of PCDs, and the role of pyroptosis in diseases has important research value and broad application prospect. Using CiteSpace and VOSviewer software for visual analysis, the research on pyroptosis is generally on the rise yearly. Globally, China and the USA are the leading countries in this research. Among the research institutions, Chinese Academy of Sciences is the institution with the highest influence on achievements. Different countries and institutions need to strengthen cooperation and exchanges. Feng Shao is an outstanding contributor to the field of pyroptosis. Most of the articles concerning pyroptosis are cited from internationally influential journals, indicating that pyroptosis has received much attention. At present, the research on pyroptosis mainly focuses on its mechanism molecules, the crosstalk of different types of PCD and its role in diseases, which will also be the focus of future research.

## Data Availability Statement

The original contributions presented in the study are included in the article/supplementary material. Further inquiries can be directed to the corresponding authors.

## Author Contributions

DM and SL conceived the study. BY, BG, and LS collected the data. LZ, TW, and ZZ re-examined the data. QL and YF analyzed the data. DM wrote the manuscript. ZG, SL, and HX reviewed and revised the manuscript. All authors contributed to the article and approved the submitted version.

## Funding

This study was supported by “National natural science foundation of China” (No.82004192) and “the Fundamental Research Funds for the Central public welfare research institutes” (ZZ14-YQ-004). National natural science foundation of China (No. 82074215); CACMS Innovation fund (CI2021A00917); National Clinical Research Center for Chinese Medicine Cardiology, Xiyuan Hospital, China Academy of Chinese Medical Sciences (CI2021A00920).

## Conflict of Interest

The authors declare that the research was conducted in the absence of any commercial or financial relationships that could be construed as a potential conflict of interest.

## Publisher’s Note

All claims expressed in this article are solely those of the authors and do not necessarily represent those of their affiliated organizations, or those of the publisher, the editors and the reviewers. Any product that may be evaluated in this article, or claim that may be made by its manufacturer, is not guaranteed or endorsed by the publisher.
